# Dietary Patterns and Alzheimer’s Disease: An Updated Review Linking Nutrition to Neuroscience

**DOI:** 10.3390/nu15143204

**Published:** 2023-07-19

**Authors:** Ines Ellouze, Julia Sheffler, Ravinder Nagpal, Bahram Arjmandi

**Affiliations:** 1 Department of Plant Biotechnology, Higher Institute of Biotechnology of Beja, University of Jendouba, Beja 382, Tunisia; ie22f@fsu.edu; 2 Department of Nutrition and Integrative Physiology, Florida State University, Tallahassee, FL 32306, USA; 3 Center for Translational Behavioral Science, Florida State University College of Medicine, Tallahassee, FL 32304, USA; julia.sheffler@med.fsu.edu; 4 Center for Advancing Exercise and Nutrition Research on Aging, Florida State University, Tallahassee, FL 32306, USA

**Keywords:** Alzheimer’s disease, aging, brain health, cognitive impairment, dementia, diet, Mediterranean diet, neuroscience, neurodegenerative disorders

## Abstract

Alzheimer’s disease (AD) is a growing concern for the aging population worldwide. With no current cure or reliable treatments available for AD, prevention is an important and growing area of research. A range of lifestyle and dietary patterns have been studied to identify the most effective preventive lifestyle changes against AD and related dementia (ADRD) pathology. Of these, the most studied dietary patterns are the Mediterranean, DASH, MIND, ketogenic, and modified Mediterranean-ketogenic diets. However, there are discrepancies in the reported benefits among studies examining these dietary patterns. We herein compile a narrative/literature review of existing clinical evidence on the association of these patterns with ADRD symptomology and contemplate their preventive/ameliorative effects on ADRD neuropathology in various clinical milieus. By and large, plant-based dietary patterns have been found to be relatively consistently and positively correlated with preventing and reducing the odds of ADRD. These impacts stem not only from the direct impact of specific dietary components within these patterns on the brain but also from indirect effects through decreasing the deleterious effects of ADRD risk factors, such as diabetes, obesity, and cardiovascular diseases. Importantly, other psychosocial factors influence dietary intake, such as the social connection, which may directly influence diet and lifestyle, thereby also impacting ADRD risk. To this end, prospective research on ADRD should include a holistic approach, including psychosocial considerations.

## 1. Introduction

Dementia is one of the most prevalent illnesses among older adults, with over 55 million people worldwide suffering from dementia. According to the World Health Organization (WHO), dementia is caused by diseases and injuries that affect the brain [1]. These diseases gradually destroy nerve cells and impair brain function over time, resulting in the deterioration of cognitive functioning beyond the normal effects of biological aging. This deterioration affects memory, thinking, processing speed, and the ability to engage in activities of daily living. Consequently, dementia leads to disability and an increasing level of dependency. Moreover, dementia imposes a significant physical, psychological, social, and economic burden on caregivers, families, and society. By 2030, the global population of older adults (aged ≥ 65 years old) is projected to reach 1 billion, accounting for 12% of the total population [2]. With an aging population, the rates of neurodegenerative diseases such as Alzheimer’s disease (AD) and related dementias (ADRD) will continue to increase.

Various cross-sectional, observational, and interventional studies, as discussed in the succeeding sections and tables, have independently linked specific dietary patterns, such as the Mediterranean diet, MIND diet, and DASH diet, to a reduced or delayed incidence or symptoms of ADRD. Therefore, our aim herein is to compile current clinical evidence concerning the associations among these dietary patterns and ADRD symptomology. Additionally, we will discuss the preventive and/or ameliorative effects of these dietary patterns on the incidence and neuropathology of AD in various clinical settings and cohorts.

## 2. Alzheimer’s Disease

According to the Centers for Disease Control and Prevention, AD is the most common form of dementia [3]. It is an incurable and progressive degenerative disease that initially manifests as mild memory loss. AD is characterized by the development of senile amyloid plaques and neurofibrillary tangles, which are associated with the gradual loss of neuronal synapses and pyramidal neurons, eventually leading to progressive neurodegeneration. The neurological regions, such as the hippocampus and neocortex, are particularly affected early in the disease [4].

The first clinical symptoms of AD typically appear around 20 years after the initial structural changes in the brain [5,6]. Additionally, this disease can result in reduced glucose metabolism, ultimately leading to brain atrophy [6,7,8]. As the disease progresses, it impairs the patient’s ability to engage in conversations and respond to the environment, primarily affecting the parts of the brain responsible for thought, memory, and language [7,8]. Several factors influence AD, including non-modifiable factors, such as genetics, sex, and age, and modifiable factors, like the level of education, physical activity, sleep, diet, smoking, and alcohol consumption, in addition to potentially modifiable factors, including metabolic syndrome during middle age [7,8].

In the United States, it is projected that by 2030, 8.2 million people will be diagnosed with AD, and this number is expected to increase to 14 million by 2060 [9,10]. Currently, AD is the sixth leading cause of death among adults aged 65 or older, and the number of deaths resulting from AD continues to rise [11]. Despite the significant economic burden and extensive research efforts to find a cure, AD remains incurable. There are currently no approved drugs available that can cure or effectively reverse AD. Consequently, there is great interest in preventive actions and interventions [12]. These interventions aim to promote healthy brain aging. Given that there are currently no treatment options for ADRD pathology, the manifestation of the disparity between cognition and the magnitude of pathology indicates the significance of exploring modifiable risk factors for improving cognitive health without specifically treating the ubiquitous aging-associated neuropathology [13,14,15]. One-third of AD cases involve certain modifiable risk factors and are preventable through dietary and lifestyle modification [16]. Scientific research indicates that regular physical activity and a healthy diet may have a beneficial effect on human cognitive function, thereby reducing the risk of developing AD. In this view, these factors may be considered preventive measures against the development or progression of AD [17,18]. Understanding the pathways underlying ADRD is crucial to improving preventative and therapeutic approaches, which are public health priorities [19]. Emerging epidemiological and clinical evidence supports the relationship between diet and ADRD development [20].

Emerging evidence suggests that prudent dietary patterns are associated with slower cognitive decline and reduced ADRD risk [20,21], although the mechanisms underlying these effects remain unclear. One possible mechanism is that specific dietary constituents may influence neural resources and enhance cognitive health and resilience [22,23]. For instance, healthier dietary patterns have been linked to the homeostatic formation of hippocampal neurons, which are found to be impaired in the early stages of ADRD [24]. Thus, by strengthening/improving cognitive resilience over time, healthier dietary elements may lead to improved late-life cognitive trajectories [25,26,27]. Indeed, understanding and exploiting the biological elements of ADRD risk factors (such as dietary factors) may inform the development of novel interventions for disease prevention.

## 3. Methodology

This narrative review encompasses clinical trials (longitudinal, cross-sectional, prospective cohorts, interventions) and meta-analyses on middle-aged and older adult populations diagnosed with or at higher risks of developing ADRD. A comprehensive literature search was conducted using various databases, including Pubmed, Medline, ScienceDirect, and Google Scholar (last accessed on 20 May 2023). The search utilized specific keywords, such as diet, dietary pattern, Mediterranean, DASH, MIND, ketogenic, modified ketogenic, vegetarian, vegan, Alzheimer’s, Alzheimer’s disease, cognitive impairment, and dementia. The search was further refined to include only English-language studies and human studies that incorporated a meta-analysis of clinical trials and cross-sectional studies. The primary aim of this review is to evaluate current evidence on dietary patterns and their effects on ADRD risk and progression.

## 4. Impact of Specific Dietary Patterns on ADRD Progression

The Western diet is known for its high content of refined grains, sugar, unhealthy fats, and salt while having an extremely low consumption of fruits and vegetables [28,29]. These components, both individually and collectively, have been linked to the obesity epidemic, cardiovascular diseases, cancer, osteoporosis, autoimmune diseases, type 2 diabetes, and other illnesses [30,31,32]. Another characteristic of the Western diet is the high consumption of ultra-processed foods and beverages. Based on the NOVA classification system, these products are characterized by high levels of hydrogenated and/or esterified oils, added sugars, carbohydrates, saturated fats, and various additives that enhance their palatability [33]. As a result, these nutritionally poor food products often lack fiber and can disrupt the gut microbiome, leading to immune alterations [34,35]. These immune system disturbances can eventually contribute to chronic inflammation [28,29]. Moreover, the excessive consumption of foods that have a high glycemic index, are rich in saturated fats, and have high sodium content may contribute to conditions such as hypercholesterolemia and insulin resistance, which impair vasoreactivity, hemodynamic function, and endothelial integrity, thereby impeding cerebral perfusion [36]. Vascular dysfunction has long been recognized as a contributing factor to ADRD [37].

Several studies have established a causal relationship between the Western diet and pathological brain aging [38,39]. Furthermore, the Western diet has been associated with poorer cognitive function, particularly at an older age [40,41]. Therefore, for overall health and well-being, the American Heart Association and the U.S. government’s Dietary Guidelines for Americans recommend a diet centered around plant-based foods, including fruits, vegetables, whole grains, nuts, and seeds, while also incorporating fish, low-fat dairy products, and lean meat. It is advised to limit or avoid red meat, sodium, saturated fats, sugar, and highly processed foods. This dietary pattern is associated with good cognitive health [10]. The diet has been suggested to play an important role, both directly and indirectly, in cognitive health and the development of dementia [42]. Nutrients found in the diet, such as vitamins, antioxidants, and fiber, can directly impact cognitive health through their antioxidative, anti-inflammatory, and endothelial and mitochondrial functions. These nutrients also have indirect effects, as they act on the cardiovascular-related effects of diabetes, dyslipidemia, hypertension, obesity, and/or homocysteine levels [10]. As these nutrients are consumed as part of a dietary pattern, it is crucial to explore the holistic effects of these dietary patterns on health [43]. A diet comprises all the individual foods and beverages consumed on a daily basis, but dietary patterns are the outcome of an individual’s dietary history, sociocultural identity, and demographic characteristics [10]. However, only a limited number of randomized controlled trials conducted to date have assessed the effects of specific foods or dietary patterns on cognitive health, particularly in relation to ADRD [44].

**Figure 1:**
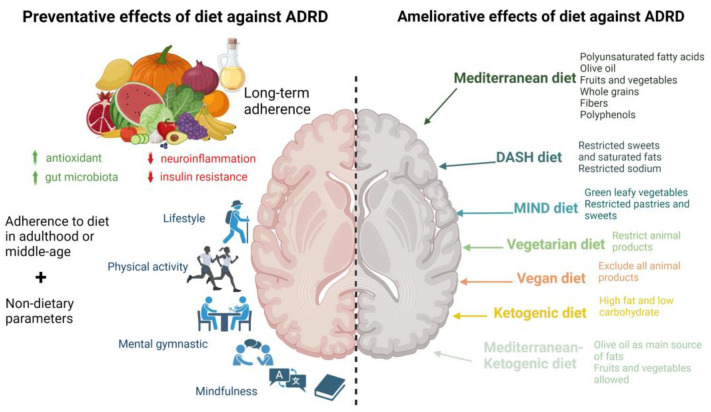
Preventive and ameliorative effects of dietary patterns against ADRD. Long-term adherence to prudent dietary patterns (**left-side panel**), in conjunction with a healthy lifestyle, may prevent ADRD via antioxidant/anti-inflammatory mechanisms and positive microbiome modulation, which are associated with lower predisposition to aging-associated neuroinflammation and insulin resistance, leading to improved lifestyle, cognitive abilities, and overall quality of life. Specific dietary patterns (**right-side panel**) may also ameliorate ADRD via different mechanisms, such as antioxidant/anti-inflammatory/microbiome-modulatory, attributed to specific macro- and micronutrients in these dietary patterns. ADRD: Alzheimer’s disease and related dementias; DASH: Dietary Approaches to Stop Hypertension dietary pattern; MIND: Mediterranean-DASH Intervention for Neurodegenerative Delay dietary patterns; ↑ higher; ↓ lower.

Hereafter, the different dietary patterns studied and their impacts on ADRD progression are presented (Figure 1).

### 4.1. Impact of Mediterranean Diet on ADRD Progression

The Mediterranean diet (MedD), created by an American couple in the 1960s, is inspired by the eating habits of people in Greece, Southern Italy, and Spain in the 1940s and 1950s [45]. These habits involve a high consumption of olive oil, unrefined cereals, fruits, and vegetables; a moderate to high consumption of fish; a moderate consumption of dairy products (mostly cheese and yogurt); moderate wine consumption; and a low consumption of red meat products [12]. The MedD consists of nutrient-dense foods that have been recognized as beneficial for overall health and healthy brain aging [9]. It has been associated with a lower risk of conversion from mild cognitive impairment to dementia [46,47,48,49], a reduction in dementia incidence [46,50,51,52,53,54,55,56], the maintenance of brain health [20,57], a lower risk of cognitive decline [41,58,59,60,61,62,63], and better cognition [64,65]. However, some inconsistencies are observed among studies regarding the benefits of the MedD on cognition and cognitive aging, particularly with regard to ADRD. Some studies did not find significant associations between the MedD and a decrease in ADRD incidence and/or conversion from mild cognitive impairment to dementia, which may be attributed to recruiting participants from outside the Mediterranean area [21,56,66,67,68,69,70,71,72,73,74]. There is evidence that the benefits of the MedD may be more prominent among individuals who have adhered to the MedD throughout their lives and reside in the Mediterranean region compared to those who adopt this dietary pattern later in life [75]. Another factor contributing to the contradictory results is the variation in the qualitative and quantitative food components prescribed in different studies. For example, some studies grouped all polyunsaturated fatty acids together, while others focused solely on olive oil. Additionally, the quantities of different components of the diet vary among studies. A randomized study, for instance, examined the impact of the MedD supplemented with olive oil or nuts and identified a significant improvement in global cognition and/or specific cognitive domains among a Spanish population with cardiovascular risk factors over a period of 6.5 years [76]. Furthermore, there is considerable variation in the scoring systems used across different studies, which can lead to disparities in estimating nutrient intake and compliance with the MedD [77]. It is important to consider potential sources of discrepancies in research findings, which may stem from differences in the studied populations, including factors such as gender (males or females only), the presence of obesity or cardiovascular diseases, and the duration of the studies. The timing of dietary adherence, whether it occurs during midlife or late life, also needs careful consideration. Table 1 provides a compilation of observational, longitudinal, and intervention studies conducted in the past 10 years examining the MedD and its association with ADRD risk factors.

In addition to cognitive outcomes associated with ADRD risk, recent research has examined the effect of the MedD on AD biomarkers, specifically β-amyloid and phosphorylated tau tangles. In a systematic review and meta-analysis conducted by Hill et al. [78], it was concluded that adherence to a Mediterranean-style dietary pattern was associated with a reduction in AD biomarkers (β-amyloid and tau tangle) and subsequent pathology. Specifically, in the latest published study by Agarwal et al. [79], which involved autopsied older adults, higher scores of adherence to the MedD were significantly associated with lower global AD pathology (*p* = 0.039). Even after adjusting the models for other covariates, such as physical activity, smoking, and vascular disease burden, the association remained significant (*p* = 0.027). When excluding the dietary assessments from the last year of life of the participants to account for events that might have altered their diet, greater overall adherence to the MedD for almost a decade remained significantly associated with reduced global AD pathology and β-amyloid load.

**Figure 2:**
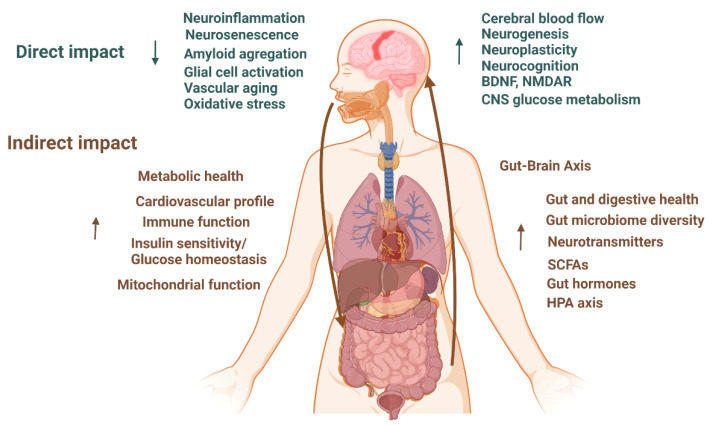
Hypothesized direct and indirect impacts of dietary patterns on ADRD [12,46,47,48,49,50,51,52,53,54,55,56,85,86,93,99,100,101,102,103]. BDNF: Brain-Derived Neurotrophic Factor; NMDR: N-methyl-D-aspartate receptor; CNS: central neural system; SCFAs: short-chain fatty acids; HPA: hypothalamic–pituitary–adrenal; ↑ higher; ↓ lower.

Furthermore, the MedD has shown potential in reducing the incidence of ADRD through its beneficial effects on blood pressure [80], mitochondrial structure and function [81], the preservation of white matter microstructure [82,83], the induction of cerebral blood flow [81], cortical thickness [84], and the accumulation of white matter hyperintensities [85]. The MedD can also act through a variety of mechanisms, including anti-inflammatory, antioxidant, and lipid-lowering actions [86,87], as well as its favorable impact on cardiovascular risk factors [87,88,89]. MedD has also been associated with higher total brain volume and cortical thickness and lower white matter hyperintensities [84,90,91,92,93]. All these factors can contribute to reducing ADRD (Figure 2).

It should be noted that adherence to the MedD may come with higher financial costs compared to other diets, as reported in studies conducted in the UK and Spain that highlighted this aspect [94,95]. Therefore, individuals with higher incomes have a higher likelihood of adhering to the MedD [96]. Future studies focusing on the MedD should consider the participants’ socioeconomic status as a covariate for dietary analysis [75], as well as other lifestyle factors, such as social contact and physical activity [97,98].

**Table 1:** A summary of clinical studies examining Mediterranean diet impact on ADRD.

Study Design	Country	Population	Follow-Up	Exposure	Outcome	Results	Covariates	Reference
Longitudinal	US	Older adults in CCMS Age: ≥65 years n = 3580	10.6 years	142-item FFQ, MedD score, DASH score, global cognition (3 MS)	Associations between DASH and MedD diets and age-related cognitive change.	Higher quintile of MedD score associated with better average cognition during follow-up but not with cognitive function rate of change.	Age, gender, education, BMI, frequency of moderate physical activity, multivitamin and mineral supplement use, history of drinking and smoking, and history of diabetes, heart attack, or stroke.	[104]
Longitudinal	US	Participants in United States Reasons for Geographic and Racial Differences in Stroke study n = 17,478 (7548 M 9930 F) Age: 64.4 years	4 years	98-item block FFQ, MedD score, cognitive impairment, six-item screener (SIS)	Higher adherence to MedD and likelihood of incident cognitive impairment (ICI) and the interaction of race and vascular risk factors.	High compared with low adherence to MedD significantly associated with lower risk of ICI. Higher tertile of MedD score significantly associated with lower risk of ICI.	Age, gender, race, region, educational level, income, number of packs smoked per year, weekly exercise, diabetes mellitus, hypercholesterolemia, atrial fibrillation, history of heart disease, BMI, waist circumference, systolic and diastolic blood pressure, ACE inhibitors/angiotensin receptor blockers, β-blockers, other antihypertensive medication, depressive symptoms, and self-reported health status.	[105]
Longitudinal	Sweden	Senior participants in Prospective Investigation of the Vasculature in Uppsala Seniors Age: 70.1 ± 0.01 years at enrollment n = 194 (101 M 93 F) cognitive assessment at 75 years	5 years	7-day food diary, adapted MedD score, dietary components, global cognition (7 MS), brain volume (3D T1-weighted MRI scan)	Association between dietary habits, cognitive functioning, and brain volumes in older individuals.	Continuous MedD score not significantly associated with global cognitive function after adjustment. Continuous MedD score not associated with gray or white matter volume or total brain volume.	Gender, energy intake, education, self-reported physical activity, low-density cholesterol, BMI, systolic blood pressure, and HOMA-IR.	[99]
Longitudinal	US	Subset of participants from the Women’s Health study n = 6174 (0 M 6174 F) Age: 72 years	4 years	131-item SFFQ, adapted MedD score, dietary components, global cognition (TICS, EBMT, CF) and verbal memory (EBMT, delayed recall of TICS 10-word list)	Association of adherence to MedD with cognitive function and decline.	MedD score quintile not significantly associated with better average global cognition or verbal memory nor with change in global cognition and verbal memory.	Treatment arm, age at initial cognitive testing, Caucasian race, high education, high income, energy intake, physical activity, BMI, smoking, diabetes, hypertension, hypercholesterolemia, hormone use, and depression.	[67]
Longitudinal	US	Women from the Nurses’ Health Study n = 16,058 (0 M 16,058 F) Age: 74.3 years	6 years	116-item SFFQ, adapted MedD score, dietary components, global cognition (TICS and composite score of TICS, EBMT, CF, DST backward), and verbal memory (immediate and delayed recalls of the EBMT and TICS)	Associations between long-term adherence to MedD and subsequent cognitive function and decline.	Long-term higher quintile MedD score at older age significantly associated with better performance on TICS, global cognition, and verbal memory. Quintile of average MedD score not significantly associated with change in TICS score, global cognition, or verbal memory.	Age, education, long-term physical activity and total energy intake, BMI, smoking, multivitamin use, and history of depression, diabetes, hypertension, hypercholesterolemia, or myocardial infarction.	[54]
Longitudinal	US	Subset of participants from The Supplementation with Vitamins and Mineral Antioxidants study n = 3083 (1655 M 1428 F) Age: 52.0 ± 4.6 years at enrollment	13 years	24 h food recalls (12: each year), MedD score, Mediterranean-Style Dietary Pattern Score (MSDPS), cognitive performance (episodic memory, lexical-semantic memory, short-term memory, working memory, mental flexibility	Association between midlife MedD adherence and cognitive performance assessed 13 years later.	Higher tertile of MedD score associated only with working memory span. Higher tertile of MSDPS significantly associated with semantic fluency on the phonemic fluency task, but not with global cognition, episodic memory, short-term memory, working memory, or mental flexibility.	Age, gender, education, follow-up time, supplementation group during the trial phase, number of 24 h dietary records, total energy intake, BMI, occupational status, smoking status, physical activity, memory difficulties at baseline, depressive symptoms concomitant with the cognitive function assessment, and history of diabetes, hypertension, or CVD.	[55]
Longitudinal	Italy	Subset of participants in TRELONG Study n = 309 (120 M 189 F) Age: 79.1 ± 9.65 y	7 years	FFQ, MedD yes/no (based on cereal, fish, vegetable and fruit intake), global cognition (MMSE)	Association between risk factors (body mass index (BMI), depression, chronic diseases, smoking, and lifestyles) and cognitive decline in older adults.	Adherence compared to non-adherence to MedD not significantly associated with less cognitive decline	NS	[106]
Longitudinal	US	Older participants from the Memory and Aging Project cognitively normal at enrollment n = 826 (26%M) Age: 81.5 ± 7.1 years	4.1 years	144-item FFQ, battery of cognitive tests: episodic memory, semantic memory, working memory, perceptual speed, and visuospatial ability.	Association between DASH and MedD diets and slower cognitive decline.	A 1-unit higher MedD score associated with a 0.002 slower rate of global cognitive decline standardized units, after adjustment for covariates.	Age, gender, education, participation in cognitive activities, total energy intake (kcal), time, and the interaction between time and each covariate, physical activity, presence of APOE ε4 alleles, depression, hypertension, diabetes, and stroke.	[58]
Longitudinal	Greece	Older adults in European Prospective Investigation into Cancer and Nutrition (EPIC) n = 401 (144 M 257 F) Age at enrollment: 74 years	6.6 years	150-item SFFQ, MedD score, dietary components, global cognition (MMSE)	Association between adherence to MedD in a Mediterranean country and cognitive decline in older adults.	Higher tertile of MedD scores significantly associated with less mild cognitive decline and substantial cognitive decline.	Age, gender, years of education, BMI, physical activity, smoking status, diabetes, hypertension, cohabiting, total energy intake.	[49]
Longitudinal	China	Older adults in China Health and Nutrition Survey n = 1650 (820 M 831 F) Age: 63.5 years	5.3 years	3-day 24 h recall, adapted MedD score, dietary components, decline in global cognition, composite z-scores, and verbal memory (modified TICS)	Association between cognitive changes among Chinese older adults and either an adapted Mediterranean diet score or factor-analysis-derived dietary patterns.	Higher MedD score significantly associated with slower rate of decline in global cognitive, composite z-, and verbal memory scores only in participants ≥ 65 years. Higher tertile of MedD score significantly associated with less decline in global cognitive scores and verbal memory scores only in participants ≥ 65 years	Age, gender, education, region, urbanization index, annual household income per capita, total energy intake, physical activity, current smoking, time since baseline, BMI, hypertension, and time interactions with each covariate.	[61]
Longitudinal	Sweden	Older adults in Uppsala longitudinal study n = 1038 (1038 M 0 F) Age at enrollment: 70 years	12 years	7-day food diary, adapted MedD score, AD (based on NINCDS-ADRDA and DSM-IV criteria), dementia, and cognitive impairment (MMSE)	Associations between development of cognitive dysfunctions and different diets. (Healthy Diet Indicator), a Mediterranean-like diet, and a low-carbohydrate, high-protein diet.	Continuous MedD score not associated with a lower risk of AD, dementia, or cognitive impairment. Higher tertile of MedD score not associated with AD or cognitive impairment. Highest tertile of MedD score in participants with energy intake according to the Goldberg cut-off significantly associated with a lower risk of cognitive impairment.	Energy, education, presence of APOE ε4 allele, living alone, smoking, and physical activity.	[72]
Longitudinal	US	Participants of the Rush Memory and Aging Project (MAP) n = 923 (±24% M) Age: 58–98 years	4.5 years	144-item SFFQ, A-MedD, A-DASH, and MIND scores, AD (based on NINCDS-ADRDA criteria)	Association of MIND, a hybrid Mediterranean and DASH diet, with incident Alzheimer’s disease.	Highest tertile of A-MedD adherence significantly associated with lower risk of AD diagnosis.	Age, gender, education, presence of APOE ε4 allele, participation in cognitively stimulating activities, physical activity, total energy intake, and cardiovascular conditions.	[100]
Longitudinal	US	Older adults in Health, Aging, and Body Composition (Health ABC) n = 2326 (1109 M 1217 F) Age: 70–79 years	7.9 years	108-item block FFQ via interviews, A-MedD score (race-specific), global cognition (3 MS score)	Association of decreased risk of cognitive decline with MedD within a diverse population.	Among African American, but not among whites, A-MedD score significantly associated with less cognitive decline.	Age, gender, education, BMI, current smoking, physical activity, depression, diabetes, total energy intake, and socioeconomic status.	[60]
Longitudinal	Spain	Participants in Spanish SUN project n = 823 (597 M 223 F) Age: at enrollment, 61.9 ± 6.0 years	6–8 years	136-item SFFQ, MedD score, dietary components, cognitive function (TICS)	Association between adherence to MedD and cognitive function in a Spanish population.	Lower tertile of MedD score significantly associated with faster cognitive decline.	Age, gender, presence of APOE ε4 allele, follow-up time, total energy intake, BMI, smoking status, physical activity, diabetes, hypertension, hypercholesterolemia, history of CVD, and years of university education.	[59]
Longitudinal	US	Postmenopausal women enrolled in the Women’s Health Initiative Memory Study (WHIMS) n = 6425 (0%M) Age: 65–79 years	9.11 years	FFQ, A-MedD score, DASH score, MCI (MMSE and battery of neuropsychological tests)	Association of dietary patterns with cognitive decline in older women and association of dietary patterns with risk of cognitive decline in women with hypertension.	A-MedD score quintile not significantly associated with reduced risk of MCI. Higher quintile of A-MedD score in a subset of white women with adjustment for APOE ε4 allele quintile significantly associated with a lower risk of MCI.	Age, race, education level, Women’s Health Initiative hormone trial randomization assignment, baseline 3 MS level, smoking status, physical activity, diabetes, hypertension, BMI, family income, depression, history of CVD, and total energy intake.	[73]
Longitudinal	Italy	Older adults in InCHIANTI study n = 832 (44% M) Age: 75.4 ± 7.6 y	10.1 years	FFQ, MedD score, dietary components, global cognition (MMSE)	Association between MedD and trajectories of cognitive performance in the InCHIANTI study.	Continuous MedD score and higher tertile of MedD significantly associated lower risk of cognitive decline based on MMSE.	Age, gender, study site, chronic diseases, years of education, total energy intake, physical activity, BMI, presence of APOE ε4 allele, CRP, and IL-6.	[63]
Longitudinal	Sweden	Older adults in Swedish National study on Aging and Care n = 2223 (871 M 1352 F) Age: M: 69.5 ± 8.6 and F: 71.3 ± 9.1 years	6 years	98-item SFFQ, A-MedD, A-DASH, and MIND scores, dietary components, global cognition (MMSE)	Association between slower cognitive decline and dietary patterns: MIND, DASH, MedD, and a Nordic dietary pattern.	Higher A-MedD score significantly associated with less cognitive decline. A-MedD score not significantly associated with a lower risk of MMSE score ≤24.	Total caloric intake, age, gender, education, civil status, physical activity, smoking, BMI, vitamin/mineral supplement intake, vascular disorders, diabetes, cancer, depression, presence of APOE ε4 allele, and dietary components other than those included in each dietary index.	[41]
Longitudinal	US	Male health professional participants in Health Professionals Follow-up Study n = 27,842 (27,842 M 0 F) Age at baseline: 51 y	±26 years	FFQ, MedD score, dietary components, subjective cognitive function (SCF)	Association between long-term adherence to MedD and self-reported subjective cognitive function.	Higher quintile of MedD score associated with a lower risk of both poor SCF and moderate SCF.	Age, smoking history, diabetes, hypertension, depression, hypercholesterolemia, physical activity level, BMI.	[62]
Longitudinal	Australia	Older Australian adults n= 1220 (50% men) Age: 60–64 years	12 years	CSIRO-FFQ, MedD, and MIND scores, dietary components	Cognitive impairment: MCI/dementia (Winbald criteria, NINCDS-ADRDA criteria).	Higher tertile of MedD score not significantly associated with cognitive impairment.	Energy intake, age, sex, presence of APOE ε4 allele, education, mental activity, physical activity, smoking status, depression, diabetes, BMI, hypertension, heart disease, and stroke.	[74]
Longitudinal	US	Participants in the Cognitive Reserve (CR) study and the Reference Ability Neural Network (RANN) study n = 183 (89 M 94 F) Age: 53.19 ± 16.52 years	5 years	FFQ, MedD score, brain MRI	Association of greater adherence to MedD with less accumulation of white matter hyperintensities (WMHs).	MedD adherence negatively associated with an increase in WMHs, adjusting for all covariates. Association between MedD and WMH change moderated by age.	Age, gender, and race/ethnicity.	[85]
Cross-sectional	Greece	Older adults n = 557 (237 M 320 F) Age > 65 years	NS	157-item EPIC-Greek SFFQ, A-MedD score, cognitive impairment (MMSE)	Association of dietary habits with cognitive function among seniors.	Continuous MedD score significantly associated with less cognitive impairment in men but more cognitive impairment in women.	Age, GDS, education, social activity, smoking, metabolic syndrome.	[107]
Cross-sectional	Australia	Participants from Southern Australia n = 1183 (432 M 751 F) Age: 50.6 ± 5.8 years	NS	215-item FFQ, MedD score, dietary components Self-reported cognitive function (CFQ) on mistakes in tasks, perception, memory, and motor function	Association of level of adherence to the MedD with cognitive function and psychological well-being.	MedD score not significantly associated with self-reported cognitive function.	Age, gender, education, BMI, exercise, smoking, and total energy intake.	[108]
Cross-sectional	China	Chinese older adults from Hong Kong n= 3670 (1926 M 1744 F) Age: >65 years	NS	280-item FFQ, MedD score, cognitive function (CSI-D)	Association of a priori or a posteriori diet with risk of cognitive impairment.	No significant association between MedD score and cognitive function in men and women.	Age, BMI, PASE, energy intake, education level, Hong Kong community ladder, smoking status, alcohol use, number of ADLs, GDS category, and self-reported history of diabetes, hypertension, and CVD/stroke.	[66]
Cross-sectional	Scotland	Participants enrolled in 1936 n = 878 (±50% M) Age: 69.5 years	NS	168-item FFQ, MedD (22 items), cognitive function (IQ (MHT), general cognition (WAIS-III LNS, MR, BD, DS, DST backward, SS), processing speed (SS, DS, SCRT, IT), memory LM and VPA immediate and delayed recalls, SSP forward and backward, LNS, DST backward, and verbal ability (NART, WTAR))	Association between dietary patterns and better cognitive performance in later life, taking into consideration childhood intelligence quotient (IQ) and socioeconomic status.	MedD score positively associated with verbal ability only.	Age, gender, occupational social class, IQ at age of 11 years.	[109]
Cross-sectional	Poland	Older adults with high risk of metabolic syndrome n = 87 (31 M 56 F) Age: 70.0 ± 6.5 years	NS	FFQ, A-MedD score (high vs. low), dietary components, MCI, global cognition (MMSE), attention (TMT), visual memory (PRM), executive function (ST, SOC, SWM, SSP)	Association between adherence to MedD and cognitive function (CF), along with selected sociodemographic (SD) and clinical indices.	High MedD score significantly associated with lower prevalence of MCI and higher global cognition, but not with attention, visual memory, or executive function.	Gender, age, education level, smoking status, family status, leisure time physical activity, and existence of metabolic syndrome.	[48]
Cross-sectional	US	Participants in study of aging and dementia WHICAP n = 674 (220 M 454 F) Age: 80.1 ± 5.6 years	NS	FFQ, MedD score, MRI, total brain volume (TBV); total gray matter volume (TGMV); total white matter volume (TWMV), cortical thickness	Association of higher adherence to a MedD diet with larger MRI-measured brain volume or cortical thickness.	MedD adherence associated with less brain atrophy, with an effect similar to 5 years of aging.	Age at time of scan, gender, ethnicity, education, BMI, diabetes, mean cognitive z-score, presence of APOE ε4 allele, caloric intake, hypertension, heart disease, and stroke.	[90]
Cross-sectional	US	Older adults n = 5907 (40% men) Age: 67.8 years	NS	Cognitive performance (global cognition score based on immediate and delayed recall, backward counting, and serial seven subtraction)	Association between the MedD and MIND diets and cognition in a nationally representative population of older U.S. adults.	Higher tertile of A-MedD score significantly associated with better cognitive performance and lower risk of poor cognitive performance.	Age, gender, race, low education attainment, current smoking, obesity, total wealth, hypertension, diabetes mellitus, physical inactivity, depression, and total energy intake.	[65]
Cross-sectional	US	Older Spanish adults n = 79 (36 M 41 F) Age: 81.0 years	NS	3-day 24 h diet recalls and a face-to- face interview, 14-item Mediterranean Diet Adherence Screener (MEDAS), global cognition (MMSE)	Association of adherence to MedD and cognitive status and depressive symptoms in older adults.	Higher tertile of MEDAS score significantly associated with better cognitive status.	NS	[64]
Cross-sectional	Greece	Older adults in Hellenic Longitudinal Investigation of Ageing and Diet n = 1864 (757 M 1107 F) Age: 73.0 ± 6.1 years	NS	SFFQ, A-MedD score, dietary components, cognitive status (dementia (DSM-IV, NINCDS/ADRDA criteria)) and cognitive performance (memory (GVLT), language (BNT, CIMS; categories: objects and the letter A), executive functioning (TMT, verbal fluency, months forward and backward), and visuospatial perception (TMT))	Association of adherence to an a priori defined MedD and its components with dementia and specific aspects of cognitive function in a representative population cohort in Greece.	Continuous A-MedD score and A-MedD score quartile significantly associated with lower risk of dementia. A-MedD score significantly associated with composite z-score, memory, language, and executive functioning but not with visuospatial perception.	Age, gender, education, number of clinical comorbidities, and energy intake.	[52]
Cross-sectional	US	Clinically and cognitively normal participants who were enrolled in observational brain imaging studies n = 116 (44 M 72 F) Age: 50 ± 6 years	NS	FFQ, MedD score, memory (immediate and delayed recall), executive function (WAIS), language (WAIS vocabulary), and MRI-based cortical thickness	Effects of lifestyle and vascular-related risk factors for Alzheimer’s disease (AD) on in vivo MRI-based brain atrophy in asymptomatic young to middle-aged adults.	Continuous MedD score significantly positively associated with MRI-based cortical thickness of the posterior cingulate cortex. MedD score not significantly associated with memory, executive function, or language.	Age, gender, presence of APOE ε4 allele.	[84]
Cross-sectional	US	Older participants in study focusing on healthy brain aging and cardiovascular disease risk factors n = 82 (40 M 42 F) Age: 68.8 ± 6.88 years	NS	FFQ, MedD score, cognitive assessment: information processing, executive functioning, MRI scans	Associations between MedD and cognitive and neuroimaging phenotypes in a cohort of nondemented, nondepressed older adults.	After adjustment with all covariates, a significant effect of MedD score on the volume of the dentate gyrus.	Age, gender, education, BMI, and estimated daily calorie intake.	[101]

Adapted from [20] and updated. ACE: angiotensin-converting enzyme; AD: Alzheimer’s disease; ADLs: activities of daily living; APOE: apolipoprotein E; BD: block design; BMI: body mass index; BNT: Boston Naming Test; CIMS: Complex Ideational Material Subtest; CF: category fluency; CRP: C-reactive protein; CSI-D: Community Screening Instrument for Dementia; CSIRO-FFQ: Commonwealth Scientific and Industrial Research Organization semi-quantitative food frequency questionnaire; CVD: cardiovascular disease; DS: Digit Symbol; DST: Digital Span Task; EBMT: East Boston Memory Test; FFQ: Food Frequency Questionnaire; GDS: Geriatric Depression Scale; GVLT: Greek Verbal Learning Test; IL-6: Interleukin 6; IT: Inspection Time; IQ: intelligence quotient; LM: logical memory; LNS: Letter Number Sequencing; MCI: mild cognitive impairment; MedD: Mediterranean diet; MHT: Moray House Test; MR: matrix reasoning; MRI: Magnetic Resonance Imaging; NS: not specified; NART: National Adult Reading Test; PASE: Physical Activity Scale for Elderly; PRM: pattern recognition memory; SFFQ: semi-quantitative food frequency questionnaire; SCRT: Simple and Choice Reaction Time; SOC: the Stockings of Cambridge Test; SS: Symbol Search; SSP: Spatial Span; ST: Stroop test; SWM: spatial working memory; RI: Rappel indicé; TICS: Telephone Interview for Cognitive Status; TMT: Trail Making Test; VPA: verbal paired associates; WAIS-III: Wechsler Adult Intelligence Scale-III; WTAR: Wechsler test of adult reading; 3 MS: Modified Mini-Mental State Examination; 7 MS: 7 min screening.

### 4.2. Impact of DASH Diet on ADRD Progression

The Dietary Approaches to Stop Hypertension (DASH) diet is a dietary pattern that aims at preventing and treating hypertension and improving cardiovascular health [110]. The DASH diet shares several similarities with the MedD, as both encourage a high intake of plant-based foods. However, the DASH diet also emphasizes the low consumption and/or limitation of dietary sodium, sweetened beverages, and red meats, and it does not recommend alcohol [12]. Similar to the MedD, the DASH diet has been shown to prevent several cardiovascular risk factors, including high blood pressure and LDL cholesterol, which are associated with the development of dementia and particularly ADRD. Additionally, the DASH diet can modulate oxidative stress, inflammation, and insulin resistance, which are involved in the pathological process of ADRD (Figure 2) [111]. Long-term adherence to the DASH diet has been associated with better cognitive function. For example, this association was found in older American women participating in a six-year follow-up study from the Nurses’ Health study [112], as well as in an observational study spanning 11 years involving older adult men and women in the Cache County Memory Study [104], and in participants of the Memory and Aging Project over 4.1 years, who exhibited slower rates of cognitive decline [58]. However, a cross-sectional study focusing on sedentary adults with cognitive impairment and cardiovascular disease risks reported mixed results regarding cognitive functions. While high adherence to the DASH diet was associated with better verbal memory, it had no effect on executive function, processing speed, or visual memory [102]. Another randomized trial concluded that combining the DASH diet with weight management significantly improved executive function and memory/learning (*p* = 0.008). When the DASH diet was implemented alone, it showed an improvement in psychomotor speed (*p* = 0.036) for hypertensive overweight adults in the U.S. after a 4-month intervention [103]. In the same study, when combining the DASH diet intervention with weight management, greater improvements were observed in executive function, memory, learning (*p* = 0.008), and psychomotor speed (*p* = 0.023).

Discrepancies have been reported, as other studies have not found a significant association between the DASH diet and cognition. For instance, a prospective longitudinal study among older women in the Women’s Health Initiative Memory Study by Haring et al. [73] concluded that the DASH diet had no significant association with cognitive decline, nor did a high DASH score have an impact on cognitive decline in older community participants (both men and women) in other longitudinal studies [41,113,114]. Another six-month randomized controlled trial with sedentary men and women concluded that the DASH diet alone did not improve cognitive impairment (*p* = 0.059), but when combined with aerobic exercise, a considerable improvement in executive function (*p* = 0.012) was observed [115]. These results suggest that the DASH diet, when combined with other non-dietary interventions, possibly synergistically, provides a neuroprotective effect. Table 2 summarizes the different clinical trials investigating the relationship between the DASH diet and ADRD and cognition in the last 10 years. The observed discrepancies in Table 2 may be attributed to differences in study design (RCT vs. observational or cross-sectional), the number and variety of participants (e.g., men only, specific age group, obese or lean individuals), the scoring systems used to define adherence to the DASH diet, and the food products considered in each study. Overall, while the existing evidence hints that the DASH diet might be beneficial for cognitive functioning, further research is needed to validate these findings and demonstrate the benefits of DASH for ADRD progression and risk.

### 4.3. Impact of MIND Diet on ADRD Progression

The MIND diet, which stands for Mediterranean-DASH Intervention for Neurogenerative Delay, combines elements from both the Mediterranean and DASH diets, with a specific focus on dietary components with neuroprotective effects [12]. Unlike the Mediterranean diet, the MIND diet emphasizes the consumption of berries and green leafy vegetables rather than a high intake of fruits. Numerous observational studies and clinical trials have examined the impact of the MIND diet on ADRD progression (Table 3), and all of these studies suggest a positive association with better cognition [20,41,100,116,117,118,119], lower risks of cognitive impairment [74], and a reduced risk of developing AD [100]. An observational study conducted by Morris et al. [100] over an average of 4.7 years highlighted that high adherence to the MIND diet was associated with less cognitive decline compared to low adherence (*p* < 0.0001), suggesting that the MIND diet may slow the rate of cognitive decline. Another cross-sectional study involving older U.S. citizens found that higher adherence to the MIND diet was associated with improved cognitive function in a dose–response manner (*p* < 0.001) [65]. Long-term adherence to the MIND diet was associated with moderately improved verbal memory in later life over a 12.9-year follow-up [120], and a longitudinal study with a 12-year follow-up concluded that the MIND diet reduced the risk of cognitive decline by 53% [74]. Additionally, a study on older adults with a 6-year follow-up on MIND diet adherence showed that a one-point increase in the MIND diet score was associated with a 14% reduction in the risk of subjective memory complaints [121]. Furthermore, a study on older adults highlighted a strong and significant association between MIND diet adherence and better cognitive functioning, even among those diagnosed with AD before or after death [122]. A systematic search conducted by Chu et al. [12] concluded that higher adherence to the MIND diet may be associated with a lower incidence of cognitive impairment and AD, while the MedD appears to provide greater neuroprotection against ADRD. Huang et al. [123] attempted to establish a Chinese version of the MIND diet that aligned with Chinese dietary characteristics and culture while also being more affordable. Moderate and high adherence to the developed Chinese MIND diet were both associated with lower odds of cognitive impairment and IADL disability (0.81 and 0.6, respectively), even after adjusting for covariates. In a recent study by Agarwal et al. [79], higher MIND diet scores were negatively correlated with lower global AD pathology (*p* = 0.047), and this association remained significant even after adjusting for other covariates (*p* = 0.047). Participants who had a one-unit higher MIND diet score showed amyloid loads similar to those of individuals who were four years younger [79]. This effect persisted even after adjusting for other lifestyle factors and the burden of vascular disease. A randomized controlled trial conducted over 3 months with a MIND diet intervention for postmenopausal women demonstrated a significant improvement in working memory and verbal recognition [124]. However, combining the MIND diet with different lifestyle components, particularly physical activity, may be more effective in slowing ADRD progression when diagnosed early, as indicated by a cross-sectional study involving both physical activity and the MIND diet in an older adult population [125]. Overall, the current evidence indicates that the MIND diet may be associated with a reduced risk of ADRD and slowed ADRD progression.

### 4.4. Impact of Ketogenic Diet on ADRD Progression

The ketogenic diet is characterized by high fat and low carbohydrate intake, which promotes ketone production for energy [12]. This diet has been successfully used to treat patients with refractory epilepsy [129], and growing evidence indicates that it may have benefits for cognitive functioning. In a recent case study conducted by Morrill and Gibas [130], the ketogenic diet was found to increase cognitive assessment scores in ApoE4-positive patients with mild AD. Chu et al. [12] extensively reviewed human studies linking the ketogenic diet to cognitive impairment and ADRD development. Out of the 15 identified studies, 14 indicated a significant improvement in cognitive function when ketogenic diets or ketone supplements were administrated to patients with mild cognitive impairment, mild-to-moderate AD, or AD. Grammatikopoulou et al. [131] identified 10 randomized controlled trials that focused on the impact of ketogenic therapies on improving cognitive function and delaying AD progression. The beneficial effects of the ketogenic diet were observed both after acute consumption and with long-term adherence, particularly in participants with mild cognitive impairment. One characteristic of the ketogenic diet is its level of restrictiveness compared to other ‘healthy’ diets, such as the Mediterranean, MIND, and DASH diets [12]. Individuals adhering to the ketogenic diet often experience adverse gastrointestinal events and hypoglycemic episodes during the initial phase [132]. Additionally, long-term compliance with the ketogenic diet presents challenges. For instance, for older adults with mild cognitive impairment or established ADRD health conditions, a drastic shift toward a high-fat dietary pattern can have detrimental effects on their cardiovascular health [12,130]. The potential benefits identified in current studies suggest that more research is needed to evaluate the long-term benefits and side effects, as well as research addressing adherence to this more restrictive eating pattern. It is important to note that, to the best of our knowledge, there are no large-scale RCTs examining ketogenic diet and cognition, so more research is needed in this area.

### 4.5. Impact of Modified Mediterranean-Ketogenic Diet on ADRD Progression

To meet the dietary requirements of the late-middle-aged population and mitigate the potential negative consequences of long-term adherence to the ketogenic diet, Taylor et al. [133] proposed making the ketogenic diet nutritionally dense. This led to the development of a new diet called the modified Mediterranean-ketogenic diet (MMKD), which combines key elements of the Mediterranean and ketogenic diets. The target macronutrient composition of the MMKD is approximately 5–10% carbohydrate, 60–65% fat, and 30% protein as a percentage of total caloric intake [134]. The diet encourages the consumption of protein sources low in saturated fats, such as fish and lean meats, along with healthy fats, with a particular emphasis on extra virgin olive oil as the main source of fats. It also promotes the intake of fruits, vegetables, and whole grains within certain limits and allows for the consumption of one glass of wine per day [135]. Since the MMKD is relatively new, a few studies have investigated its relationship with AD progression (Table 4). In a pilot study, Nagpal et al. [136] highlighted that the MMKD can modulate the gut microbiome and metabolites, which are associated with improved AD biomarkers in the cerebrospinal fluid. However, they reported an increase in cerebrospinal fluid Aβ42 but a decrease in tau at the end of the 6-week intervention. Similar observations were made in a randomized controlled trial conducted by Neth et al. [135]. A 12-week intervention with MMKD resulted in improved cognitive function and everyday functioning [137]. In a crossover trial investigating the impact of MMKD, a decrease in adiposity was found to be correlated with a similar decrease in cerebrospinal fluid biomarkers [134]. The latest review conducted by Wang et al. [138] addresses different diet patterns and individual food product intake and their respective impacts on ADRD. However, conducting more studies with a large number of participants will help uncover the hidden mechanisms and better understand the impacts of MMKD on individuals with mild cognitive impairment or ADRD.

### 4.6. Impact of Vegetarian Diet on ADRD Progression

A vegetarian diet is not just a plant-based diet that restricts animal products but is also characterized by a low consumption of saturated fat and a high intake of vegetables, fruits, whole grains, legumes, nuts and seeds, dairy products, and/or eggs [139,140]. The extent to which this diet includes discretionary foods, such as sugar-sweetened beverages, desserts, and potato chips, determines its classification as a ‘healthy’ plant-based food pattern [141]. The vegetarian diet has been associated with a lower risk of several chronic diseases and may have the potential to reduce the risk of cardiometabolic and neurodegenerative diseases (Figure 2) [142]. Despite the increasing number of individuals globally adopting a vegetarian diet, only a few studies have specifically investigated the relationship between this dietary pattern and cognitive impairment, particularly ADRD (Table 5). The Adventist Health Study-2 included older adults who followed a vegetarian or non-vegetarian diet. This cohort demonstrated that vegetarians had higher adherence to their dietary patterns constantly over decades, which was associated with better memory and language abilities [143]. Liu et al. [141] assessed the difference in cognition among different races and dietary patterns. They found that a healthy plant-based diet was associated with significantly slower rates of decline in global cognition (*p* = 0.032), perceptual speed (*p* = 0.04), and episodic memory (*p* = 0.04), specifically in African American participants. A large study conducted in Taiwan concluded that vegetarians had a reduced risk of developing dementia compared to non-vegetarians [144]. Despite the limited number of studies on vegetarian diets, existing evidence suggests that such diets may have a beneficial impact on slowing the progression of cognitive decline. The vegetarian diet is rich in nutrients known for their anti-inflammatory and antioxidant activities. However, it is important to note that patients with cognitive impairment and ADRD may still require a certain intake of animal-derived products, especially meat [145,146]. Indeed, older adults have high protein needs, which will present a challenge when restricting animal product consumption to adhere to a vegetarian diet. These products ensure an important supply of B vitamins that are highly and strictly related to AD development and progression [146].

### 4.7. Impact of Vegan Diet on ADRD Progression

What distinguishes the vegan diet from plant-based and vegetarian diets is that it excludes all animal products, not just meat. In fact, the vegan diet is not only free of meat but also eliminates all animal-derived products. When compared to an omnivorous diet, the vegan diet is richer in fiber, polyunsaturated fatty acids, and vitamins [147,148,149]. These individual components can potentially slow down AD pathophysiology. Despite the suspected beneficial impacts on AD, no studies have been conducted to investigate the specific impact of a vegan diet. However, it is important to pay special attention to the long-term effects of a vegan diet, as it may lead to deficiencies in essential micronutrients such as vitamin B12 and vitamin D. These deficiencies are closely associated with the development and progression of ADRD [150].

## 5. Impact of Specific Dietary Patterns on ADRD Prevention

The multiple dietary patterns reviewed here have shown potential for slowing the progression of ADRD and may also play a preventive role. However, it is important to determine when to start adhering to these dietary patterns, given that ADRD symptoms may manifest decades after the onset of brain changes. Current research evidence suggests that the first prodromal symptoms, such as changes in the synapse number, nutritional status, cognitive level, and neuropathological changes in the central nervous system, appear around the age of 50 years. Therefore, the ideal age to start taking preventive actions and implementing diet interventions seems to be around 50–60 years old or earlier, with impacts being assessed starting from 70 years old. This is due to the prevalence of long-term interventions rather than the acute effects of consumption on ADRD prevention. As a result, most proposed research studies target participants between the age of 50 and 70 years who do not show any symptoms of cognitive impairment but carry one or several risk factors. Some researchers recommend that dietary interventions last 4–8 years to enable long-term adherence effects to accumulate [151]. To make dietary patterns effective in preventing neurodegenerative diseases, the interventions need to be balanced and meet the dietary requirements of the target population. They should help maintain proper blood pressure and optimal cholesterol, glucose, and homocysteine levels. The proposed diet should also prevent overweight and obesity, which are increasing risk factors for AD [152]. The following sections present the dietary patterns that have been studied and linked to ADRD prevention (Figure 1 and Figure 2).

### 5.1. Impact of Mediterranean Diet on ADRD Prevention

Given the extensive research on the MedD in the management of ADRD, it is reasonable to explore whether it has the potential to prevent the onset of this disease as well. In a study conducted by Hoscheidt et al. [153], normal cognitive participants who adhered to the MedD showed a decrease in Aβ_40_ levels and a shift in the Aβ_42/40_ ratio, indicating a decreased risk of AD development. The results of an epidemiological study conducted by Andreu-Reinon et al. [87] suggest that over a 20-year follow-up period, participants with high adherence to the MedD had a 20% lower risk of dementia compared to those with low adherence. In fact, every 2-point increase in MedD adherence provided an 8% lower risk of developing AD. However, when examining the linear or non-linear trends between adherence to MedD and AD, no statistically significant associations were reported (*p* = 0.196, *p* = 0.353). Similarly, the studies conducted by Féart et al. [154] and Hu et al. [155] found no statistically significant association between adherence to the MedD and the incidence of ADRD (*p* = 0.72). High adherence to the MedD was not associated with a decreased risk of ADRD after 4.1 years of follow-up in an older French population [154] or after 12 years of follow-up in an older Swedish population [72]. On the other hand, the results of the Washington Heights-Inwood Columbia Aging Project conducted by Scarmeas et al. [50] concluded that a one-point increase in the MedD scale decreased the risk of ADRD by 9%. The latest meta-analysis performed by Solch et al. [9] demonstrated a 32% decreased risk of developing ADRD when adhering to the MedD compared to low adherence, with low heterogeneity between studies. The observed beneficial effects of the MedD are attributed to its anti-inflammatory and antioxidant capacities, as well as its high fiber and polyphenol contents, which impact the gut microbiota (Figure 2) [156,157]. The reported discrepancies may be attributed to variations in scoring systems used to define diet adherence, differences in the selected populations’ age, and differences in risk factors for developing AD, as well as the duration of the follow-up and the lack of an AD diagnosis at the beginning of the study period. Despite the reported non-significant effects on ADRD prevention, it is still plausible that some protection is provided through the various mechanisms mentioned earlier. To arrive at evidence-based conclusions, it is imperative to conduct numerous clinical trials, particularly randomized controlled trials, that examine the impact of adherence to the MedD on various AD biomarkers and imaging. Additionally, long-term observational studies starting with a middle-aged population and following them for 12–15+ years are necessary.

### 5.2. Impact of DASH Diet on ADRD Prevention

There is only one longitudinal study focusing on adherence to the DASH diet and its implication in the development of ADRD in U.S. participants [100]. The study concluded that high adherence to the DASH diet decreases the risk of developing AD by 39%. The observed positive impacts are expected to be the result of long-time use, particularly before the first symptoms appear [158]. To reach a unanimous conclusion about the prevention of ADRD development through the DASH diet, several studies are required, with a special focus on early adherence (in the middle-aged population) and long-term follow-ups (12–15+ years).

### 5.3. Impact of MIND Diet on ADRD Prevention

Only one longitudinal study conducted in an older adult population from the Memory and Aging Project evaluated the relationship between the MIND diet and the prevention of ADRD [100]. The study observed a 53% lower risk of ADRD after a 4.5-year follow-up period. Furthermore, moderate adherence to the MIND diet resulted in a 35% decrease in the risk of developing AD. Undoubtedly, more studies need to be conducted to arrive at evidence-based conclusions regarding the potential beneficial effects of the MIND diet, including the examination of adherence beginning in middle age.

## 6. Discussion

Across the literature, there is relatively strong evidence that adherence to the MedD, DASH, MIND, and MMKD diets may reduce the risk of developing ADRD and slow the rate of decline. These diets may be particularly beneficial for cognitive health based on common factors, such as increased polyunsaturated fat, higher fiber, and nutrients and compounds that have neuroprotective properties. Multiple studies have provided evidence linking the intake of certain nutrients common in these diets to preventive or therapeutic effects on AD specifically [12]. For instance, polyphenols have shown the ability to promote cognitive well-being through several pathways. They can improve cerebral blood flow, reduce oxidative stress, decrease neuroinflammation, enhance neurogenesis, and promote neuroplasticity. [159,160,161,162]. Whole grains, through their cardiometabolic and gastrointestinal benefits, as evidenced by the gut microbiota–brain axis, also contribute to cognitive improvement [136,163]. Omega-3 polyunsaturated fatty acids, especially those derived from marine sources, as well as mono- and polyunsaturated fatty acids found in nuts, contribute to brain protection through various effects, including anti-inflammatory action, blood pressure reduction, and endothelial enhancement [164,165,166]. Vitamins, such as B9, B12, and E, have been associated with neurogenesis in adults due to their antioxidant effects [167,168,169]. Many of these nutrients operate through mechanistic pathways that are associated with reducing metabolic syndromes, such as lowering blood pressure, decreasing insulin resistance, and reducing blood sugar and triglyceride levels, resulting in enhanced cognitive health [170,171,172,173]. It is also important to address the appropriate proportions of these nutrient intakes within the suggested dietary patterns. Furthermore, upcoming randomized controlled trials should focus on assessing the intake of these elements within these dietary patterns and their impact on ADRD progression and prevention, as their beneficial effects may be associated with their combined consumption rather than their individual intake. In addition, the amounts of deleterious foods and drinks, such as highly processed meat, refined grains, refined sugars, and beverages with high refined sugar content, need to be monitored in the proposed dietary patterns to be studied, as these products have been significantly linked to an increased risk of cognitive impairment and ADRD. Consuming them alongside the proposed diet may decrease the effectiveness of the studied dietary pattern and its components.

## 7. Parameters Affecting Food Intake in Older Adults

Older adults often have unique dietary needs and preferences. The quality and quantity of their dietary intake are impacted by various parameters, such as physiological, economic, and societal factors, which can become more pronounced with age [174]. Some of these factors are personal, encompassing sociodemographic and psychosocial aspects, as well as lifestyle factors [175,176,177]. In fact, diet quality has been strongly linked to marital status and living arrangement. Living with a partner or in a community allows older adults to have some social support and share food [178]. Furthermore, emerging evidence shows that the association between dietary elements and neurocognition depends on socioeconomic status, with the effects of diet being more pronounced in people with low socioeconomic status [179,180]. At the same time, older adults are subject to some conditions impacting their food intake, namely, loss of sensory perceptions (olfaction, taste bud loss, salivation decrease), poor oral health, and particularly mastication difficulties, which represent barriers to diet variety and a healthy diet. ADRD in particular can lead to sensory changes that can also lead to reduced appetite. Further, certain medications and medical treatments can increase deficiencies of some nutrients, which must be emphasized in their diet. Likewise, most older adults suffer dexterity loss with aging, which can lead to practical challenges related to cooking and food preparation [181].

## 8. Other Parameters Important for ADRD

Conducting research to understand how to prevent AD is valuable. It is a part of the science of risk reduction, aiming to gather evidence on noteworthy lifestyle changes. According to the Centers for Disease Control and Prevention, healthy lifestyle habits such as regular exercise and blood pressure management are among the noticeable factors that can lower the risk of dementia. Similarly, adopting healthy habits that reduce the prevalence of cancer, diabetes, and heart diseases may also be beneficial in preventing cognitive decline. Given the specific nature of neurodegenerative diseases, other factors should also be taken into consideration for daily habits, such as not smoking, stress reduction, and engaging in mental and physical activities [152]. Emerging evidence highlights that three lifestyle components, namely, social, mental, and physical factors, are inversely correlated with the risk and incidence of ADRD. In this context, maintaining a lifestyle with high levels of physical, mental, and social activities stands out as one of the important factors contributing to enhancing the quality and expectancy of life. Enhanced psychosocial engagement, in turn, lowers the risk of dementia and neurocognitive impairment in ADRD [182]. Additionally, other factors are associated with cognitive impairment and ADRD, such as marital status and frequent social contacts. Maintaining frequent social contacts can be achieved through numerous activities, including participating in social activities, having a larger social network, and receiving greater social support, thus promoting social engagement [183]. These social parameters vary among individuals as personal attributes, and they are highly affected by the place of residence of older adults. In recent years, there has been a growing interest in the new concept of lifestyle medicine, which is associated with optimal brain health and healthy aging. This concept is based on six pillars, namely, consuming whole foods and following plant-based dietary patterns, maintaining physical activity, managing stress through non-pharmaceutical means, avoiding risky substances, ensuring good-quality sleep, and maintaining social connections [184]. Indeed, the Western lifestyle is characterized by a sedentary nature, with sedentarity or physical inactivity defined as engaging in less than 30 min of moderate-intensity physical activity on a minimum of 5 days per week or at least 20 min of vigorous-intensity physical activity on a minimum of 3 days per week [185]. Research has shown that leading an active lifestyle is inversely related to the risk of cognitive decline, with aerobic exercise training being the most widely studied form of physical activity in relation to neurocognitive function and aging [186,187]. Simultaneously, other studies have highlighted the importance of participating in leisure activities and how it can result in better diet quality for older adults [188]. Ultimately, it has been found that multidomain programs, which include interventions related to diet, nutrition, exercise, neurocognitive exercises, and social activities, yield better outcomes in slowing down disease progression compared to single-domain interventions [189].

## 9. Conclusions

Considering the rapidly growing number of people with or at risk of developing ADRD in the upcoming decades, it is crucial to find low-cost and effective ways to prevent and/or ameliorate the development and progression of these diseases. Diet, indeed, appears to be one of the most important modifiable factors that influence ADRD risk. Despite the discrepancies revealed in this review due to differences in study populations, associated risk factors, geographical regions, and scoring methods, plant-based dietary patterns generally show a positive correlation with a reduction in AD advancement and the lowering of its incidence. The MedD, DASH, MIND, and MMKD diets have the greatest efficacy for their neuroprotective benefits. The precise cellular and molecular pathways underlying and mediating the preventive and ameliorative effects of these diets on ADRD are still unclear and need to be elucidated through prospective clinical and mechanistic studies. For future studies, it is advisable to consider holistic approaches that not only incorporate dietary patterns but also encompass factors related to physical, social, economic, and mental health components.

## Figures and Tables

**Table 2:** A summary of clinical studies examining DASH diet impact on ADRD.

Study Design	Country	Population	Follow-Up	Exposure	Outcome	Results	Covariates	Reference
Longitudinal	US	Older adults in CCMS study aged over 65 years n = 3580	10.6 years	142-item FFQ, MeDi score, DASH score Global cognition (3 MS)	Associations between DASH and Mediterranean-style dietary patterns and age-related cognitive change.	Higher quintile compared to lower one of DASH score associated with better average cognition but not significantly associated with rate of change in cognitive function.	Age, gender, education, BMI, frequency of moderate physical activity, multivitamin and mineral supplement use, history of drinking and smoking, and history of diabetes, heart attack, or stroke.	[104]
Longitudinal	US	Older participants of Memory and Aging Project cognitively normal at enrollment n = 826 (26% M) Age: 81.5 ± 7.1 years	4.1 years	144-item FFQ, battery of cognitive tests: episodic memory, semantic memory, working memory, perceptual speed, and visuospatial ability	Association between DASH and Mediterranean diets with slower cognitive decline.	In models adjusted for covariates, a 1-unit increase in DASH score associated with slower rate of global cognitive decline, with a decrease of 0.007 standardized units.	Age, gender, education, participation in cognitive activities, total energy intake (kcal), time, and the interaction between time and each covariate, physical activity, presence of APOE ε4 allele, depression, hypertension, diabetes, and stroke.	[58]
Longitudinal	US	Participants of the Rush Memory and Aging Project (MAP) n = 923 (±24% M) Age: 58–98 years	4.5 years	144-item SFFQ, A-MeDi, -DASH, and MIND scores, AD (based on NINCDS-ADRDA criteria)	Association of MIND, a hybrid Mediterranean and DASH diet, with incident AD.	The highest tertile of DASH diet adherence significantly associated with lower risk of AD.	Age, gender, education, presence of APOE ε4 allele, participation in cognitively stimulating activities, physical activity, total energy intake, and cardiovascular conditions.	[100]
Longitudinal	US	Postmenopausal women enrolled in the Women’s Health Initiative Memory Study (WHIMS) n = 6425 (0% M) Age: 65–79 years	9.11 years	FFQ, A-MeDi score, DASH score, MCI (MMSE and battery of neuropsychological tests)	Dietary patterns associated with cognitive decline in older women. Dietary patterns modified cognitive decline risk for women with hypertension.	Higher quintile of DASH score significantly associated with lower risk of MCI.	Age, race, education level, Women Health Initiative hormone trial randomization assignment, baseline 3 MS level, smoking status, physical activity, diabetes, hypertension, BMI, family income, depression, history of CVD, and total energy intake.	[73]
Longitudinal	US	Participants from Nurses’ Health Study, n = 16,144 Age: at first cognitive assessment, 74.3 ± 2.3 years	4.1 years	116-item SFFQ, DASH, global cognition, and verbal memory (immediate and delayed recalls)	Association between long-term adherence to DASH diet and cognitive function and decline in older American women.	Higher long-term adherence to DASH diet associated with better average global cognition, verbal memory, and TICS, but not with change in global cognition, verbal memory, or TICS score during follow-up.	Age, education, physical activity, caloric intake, alcohol intake, smoking status, multivitamin use, BMI, history of depression, high blood pressure, hypercholesterolemia, myocardial infarction, and diabetes mellitus.	[112]
Longitudinal	Sweden	Older community participants n = 2223 (39% men) Age: 69.5 years	6 years	98-item SFFQ, A-MedD, A-DASH and MIND scores, dietary components, global cognition (MMSE)	Comparison of association of the different dietary patterns with cognitive decline in an older Scandinavian population.	DASH score not associated with cognitive decline nor with risk of MMSE score ≤ 24.	Age, gender, education, civil status, total caloric intake, BMI, physical activity, smoking, vitamin/mineral supplement intake, vascular disorders, diabetes, cancer, depression, presence of APOE ε4 allele, and dietary components other than those included in each dietary index.	[41]
Longitudinal	Spain	Participants in PREDIMED-Plus trial n = 6647 (52% men) Age: 65 years	2 years	143-item FFQ, MedD, DASH diet and MIND diet scoring, battery of cognitive tests: MMSE, visuospatial and visuo-constructive capacity, verbal ability and executive function, short-term and working memory	Relationship between baseline adherence to MedD, DASH, and MIND diets with 2-year changes in cognitive performance in older adults with overweight or obesity and high cardiovascular disease risk.	Higher adherence to DASH diet not associated with better cognitive function over 2 years.	Age, gender, education level, and civil status, physical activity, dietary intake, and smoking habit, BMI, personal history of hypertension, hypercholesterolemia, type 2 diabetes, and depression.	[113]
Longitudinal	US	Participants in MESA cohort from six communities n= 4169 (1965 M 2204 F) Age: 60.4 ± 9.5 years	2 years	120-item FFQ, cognitive function assessment by Cognitive Abilities Screening Instrument (CASI), Digit Symbol Coding (DSC), and Digit Span (DS)	Association between DASH diet and cognitive function in MESA cohort.	DASH diet adherence not associated with cognitive performance or any decline.	Age, gender, race/ethnicity, education, income, acculturation status, presence of APOE ε4 allele, total energy intake, BMI, smoking, Center for Epidemiological Studies Depression (CES-D) scale score, total intentional exercise, diabetes categories, diabetes medication use, antihypertensive medication use, Alzheimer’s medication use, stroke diagnosis, hypertension, use of antihypertensive medications, alcohol intake.	[114]
Cross-sectional	US	Sedentary adults with cognitive impairment and CVD risk factors n = 160 (33% men) Age: 65.4 years	NS	FFQ and 4-day food diary, A-MedD and A-DASH scores, verbal memory, visual memory, and executive function/processing speed score	The relationship of lifestyle factors and neurocognitive functioning in older adults with vascular risk factors and cognitive impairment, no dementia.	Higher adherence to DASH diet associated with better verbal memory, but not with executive function/processing speed or visual memory.	Age, education, gender, ethnicity, total caloric intake, family history of dementia, and chronic use of anti-inflammatory medications.	[102]
Randomized controlled trial	US	Sedentary older adults n = 160 Aerobic Exercise (AE) + DASH group, n = 40 (14 M 26 F), Age: 64.9 ± 6.2 years AE no DASH, n = 41 (12 M 29 F), Age: 65.8 ± 7.3 years DASH group, n = 41 (15 M 26 F), Age: 66.0 ± 7.1 years HE: Health Education group, n = 38 (12 M 16 F), Age: 64.7 ± 6.6 years	6 months	FFQ, DASH diet score, battery of tests: executive function, global executive function, cognitive and functional performance	Evaluation of independent and additive effects of AE and DASH diet on executive functioning in adults with cognitive impairments with no dementia.	Participants engaged in AE but not DASH diet demonstrated significant improvements in executive function. Combined AE and DASH diet associated with the largest improvements compared to receiving HE.	NS	[115]

Adapted from [20] and updated. AD: Alzheimer’s disease; APOE: apolipoprotein E; BMI: body mass index; CVD: cardiovascular disease; FFQ: Food Frequency Questionnaire; MedD: Mediterranean diet; MMSE: Mini-Mental State Examination; SFFQ: semi-quantitative food frequency questionnaire; TICS: Telephone Interview for Cognitive Status; 3 MS: Modified Mini-Mental State Examination.

**Table 3:** A summary of clinical studies examining MIND diet impact on ADRD.

Study Design	Country	Population	Follow-Up	Exposure	Outcome	Results	Covariates	Reference
Longitudinal	US	Older adults n = 923 (±24% men) Age: 58–98 years	4.5 years	144-item SFFQ, A-MeDi, A-DASH, and MIND scores	AD (based on NINCDS-ADRDA criteria)	Middle and high tertiles of MIND diet score significantly associated with lower risk of AD.	Age, gender, education, presence of APOE ε4 allele, participation in cognitively stimulating activities, physical activity, total energy intake, and cardiovascular conditions.	[100]
Longitudinal	US	Older adults n = 960 (25% men) Age: 81.4 years	4.7 years	144-item SFFQ, MIND diet score	Global cognition, episodic memory, semantic memory, visuospatial ability, perceptual speed, and working memory	MIND diet score significantly associated with slower ↘ in global cognition; episodic memory; semantic memory; visuospatial ability; perceptual speed; and working memory. Higher tertile of MIND diet score significantly associated with slower ↘ in global cognitive score.	Age, gender, education, participation in cognitive activities, smoking history, physical activity hours per week, total energy intake, presence of APOE ε4 allele, time, history of stroke, myocardial infarction, diabetes, hypertension, and interaction terms between each covariate and time).	[100]
Longitudinal	Sweden	Older-adult community residents n = 2223 (39% men) Age: 69.5 years	6 years	98-item SFFQ, A-MeDi, A-DASH, and MIND scores, dietary components	Global cognition (MMSE)	Higher MIND score significantly associated with less cognitive decline and lower risk of MMSE score ≤ 24.	Total caloric intake, age, gender, education, civil status, physical activity, smoking, BMI, vitamin/mineral supplement intake, vascular disorders, diabetes, cancer, depression, presence of APOE ε4 allele, and dietary components other than main exposure in each model.	[41]
Longitudinal	US	Older women n = 16,058 (0% men) Age: 74.3 years	12.9 years	116-item FFQ, MIND score	Global cognition (TICS and composite score of TICS, EBMT) and verbal memory (immediate and delayed recalls).	Higher adherence to MIND diet not significantly associated with less ↘ in global cognition, verbal memory, or TICS score.	Age, education, physical activity, caloric intake, alcohol intake, smoking status, multivitamin use, BMI, depression, and history of hypertension, hypercholesterolemia, myocardial infarction, and diabetes mellitus.	[120]
Longitudinal	Australia	Older Australian adults n = 1220 (50% men) Age: 60–64 years	12 years	CSIRO-FFQ, MeDi, A-MeDi, and MIND scores, dietary components	Cognitive impairment: MCI/dementia	Higher tertile of MIND score significantly associated with a lower risk of cognitive impairment.	Energy intake, age, gender, presence of APOE ε4 allele, education, mental activity, physical activity, smoking status, depression, diabetes, BMI, hypertension, heart disease, and stroke.	[74]
Longitudinal	US	Older adults with clinical history of stroke n = 106 (29 M 77 F) Age: 82.8 ± 7.1 years	5.9 years	144-item FFQ, neuropsychologic battery of tests/global cognitive decline: episodic memory, semantic memory, working memory, perceptual orientation, perceptual speed	Determination of whether the MIND diet is effective in preventing cognitive decline after stroke	Higher tertile compared with lowest one of MIND diet scores had slower rate of global cognitive ↘ and slower ↘ in semantic memory and perceptual speed. In continuous models, MIND diet associated with slower ↘ in cognitive function for global cognition and semantic memory.	Age, gender, education, total energy intake, presence of APOE ε4 allele, smoking, participation in cognitive and physical activities, depressive symptoms, BMI, chronic diseases.	[116]
Longitudinal	US	Population WRAP study n = 828 (268 M 560 F) Age: 57.7 ± 6.4 years, free of dementia and MCI at baseline	6.3 years	15-item questionnaire, neuropsychological battery of tests/ multidomain cognitive composite: immediate memory, delayed memory, executive function	Effect of MIND diet on multidomain cognitive composite and immediate/delayed memory and executive function composites	Higher MIND diet scores associated with slower ↘ in executive function. MIND diet not associated with PACC4, immediate memory, or delayed memory.	Age, gender, presence of APOE ε4 allele, cognitive activity, physical activity, education.	[117]
Longitudinal	Germany	Older adults from DELCODE study n= 389 (187 M 202 F), free of dementia Age: 69.4 ± 5.6 years Subjective Cognitive Decline group (SCD): n = 146 MCI group: n = 60 First-degree relatives of AD dementia patients: n = 35 Healthy controls: n = 148	NS	148-item FFQ neuropsychological battery of tests and MMSE tool/cognitive function: memory, language (verbal fluency), executive functioning, working memory, visuospatial functioning	Associations between dietary patterns and cognitive functioning in older adults free of dementia	Higher MIND diet score associated with better memory in total subjects and language functions in cognitively normal subjects. MIND diet not associated with executive functioning, working memory, or visuospatial functioning.	Age, gender, education, BMI, physical activity, smoking, total daily energy intake, presence of APOE ε4 allele.	[126]
Longitudinal	US	Decedent participants from Rush Memory and Aging Project (MAP) n = 569 (70% F), age at death 91 years	NS	Dietary data, cognitive testing proximate to death, and complete autopsy data at the time of the analyses	Examination of whether the association of MIND diet with cognition is independent of common brain pathologies	Higher MIND diet score associated with better global cognitive functioning proximate to death, even after adjustment for AD and other brain pathologies. MIND diet–cognition relationship remained significant when analysis restricted to individuals without MCI at baseline or postmortem diagnosed with AD.	Age at death, gender, years of education, presence of APOE ε4 allele, cognitive activities, and total energy intake.	[121]
Cross-sectional	US	Community-dwelling adults n = 5907 (40% men) Age: 67.8 years	NS	163-item SFFQ, A-MeDi score, MIND diet score	Cognitive performance: immediate and delayed recall, backward counting, and serial seven subtraction	Higher MIND diet scores associated with slower ↘ in executive function. MIND diet not associated with PACC4, immediate memory, or delayed memory.	Gender, age, race, low education attainment, current smoking, obesity, total wealth, hypertension, diabetes mellitus, physical inactivity, depression, and total energy intake.	[65]
Cross-sectional	Brazil	Senior participants with different cognition Control: n = 36 (7 M 29 F) Age ≥ 75 years 12% MCI: n = 30 (9 M 21 F) Age ≥ 75 years 50% AD: n = 30 (11 M 19 F) Age ≥ 75 years 63.3%	NS	98-item FFQ, neuropsychiatric battery of tests and MMSE tool/ cognitive performance: naming, incidental memory, immediate memory, learning, delayed recall and recognition	Impact of MIND diet adherence on cognitive performance for different cognitive profiles seniors	Moderate adherence to MIND diet associated with higher MMSE scores. High adherence to MIND diet associated with learning score in healthy older adults but not in MCI or AD. No association between the other cognitive variables and the MIND score.	Age, education, income, marital status, BMI, chronic diseases, having undergone nutritional care, motor or sensory impairments.	[127]
Cross-sectional	China	Older adults from the Chinese Longitudinal Healthy Longevity Study n = 11,245 (45.3% M) Age: 84.06 ± 11.46 years	2 to 3 years	12-items FFQ, Chinese MIND (cMIND)diet score	Cognitive impairment and IADL disability	Moderate and high adherence to cMIND diet associated with lower likelihood of cognitive impairment (higher MMSE score) and IADL disability, even after adjusting for covariates. Higher cMIND diet score associated with better cognitive function and IADL.	Age, gender, residence, education, BMI, diabetes, hearing impairment, hypertension, depression, smoking, drinking, exercise, and social engagement.	[123]
Cross-sectional	US	Data from University of Michigan Health and Retirement Study n = 3463 Age: 68.0 ± 10.0 years	NS	163-item FFQ, MIND diet scores, episodic memory (im- mediate and delayed recall), working memory, attention/processing speed	Association of combination of high-intensity physical activity (PA) and MIND diet with better cognition compared with PA or MIND diet alone or neither	MIND diet alone associated with better global cognition and lower likelihood of cognitive decline. Combining PA and MIND diet predicted better global cognition and lower likelihood of cognitive decline, but did not predict lower odds of cognitive decline compared to PA alone.	Age, gender, race, education, annual income, smoking history, hypertension, diabetes mellitus, depression, and obesity.	[125]
Randomized controlled trial	Iran	Healthy obese women (MMSE = 24) n = 37 MIND group: n = 22 Age 48.95 ± 1.07 years Control group: n = 15 Age 48.86 ± 1.56 years	3 months	168-item FFQ and 3-day food recall, neuropsychological test battery and MMSE tool/verbal short memory, working memory, attention and visual scanning, verbal recognition memory, executive function and task switching, ability to inhibit cognitive interference	Impact of 3 months MIND diet on body composition and cognition	MIND group compared with control had improved working memory, verbal recognition memory, and attention. ↗ in the surface area of inferior frontal gyrus in MIND diet group.	Pregnancy, metabolic complications, severe untreated medical, neurological, psychiatric diseases, or gastrointestinal problems.	[124]
Prospective cohort	Spain	Older adults from “Seguimiento Universidad de Navarra” n= 806 (562 M 244 F) without cognitive impairment at baseline Age: 61 ± 6 years	6 ± 3 years	136-item FFQ, telephone-based interview of MMSE/Spanish TICS (orientation, memory, attention/calculation, and language)	Compare association of dietary patterns with cognitive function	Higher adherence to MIND diet associated with upward 6-year changes in Spanish TICS scores. Each 1-point ↗ in the MIND score ↗ Spanish TICS score by 0.27 points. In adjusted linear mixed model, MIND diet score associated with 0.038 rate of change in STICS scores over 5.6 years.	Age, gender, presence of APOE ε4 allele, smoking, education, total energy intake, physical activity, BMI, alcohol intake, depression, chronic diseases, high cholesterol, low HDL cholesterol.	[128]
Prospective cohort	France	Older Adults in NutriNet-Santé n = 6011 (2384 M 3627 F) without SMC at enrollment	6 years	FFQ, SMC measured with French version of the validated self-administered cognitive difficulties scale	Impact of MIND diet adherence on SMC	MIND diet score not significantly associated with SMC in adults aged 60–69 years. Adherence to MIND diet for >70 years without depressive symptoms associated with SMC. One-point increase in MIND diet score associated with 14% ↘ in SMC risk.	Age, gender, marital status, education, occupation, smoking, income, BMI, physical activity, depressive symptoms, chronic diseases, energy intake.	[121]
Prospective cohort	US	Older population of Framingham Heart Study n = 2092 (956 M 1036 F) Age: 61 ± 9 years	6.6 ± 1.1 years	FFQ, neuropsychological testing, and brain MRI scans (n = 1904).	Association between the MIND diet and measures of brain volume, silent brain infarcts (SBIs), or brain atrophy in the community-based Framingham Heart Study	Higher MIND diet scores associated with better global cognitive function, verbal memory, visual memory, processing speed, and verbal comprehension/reasoning and with larger total brain volume following adjustments. Higher MIND diet scores not associated with other brain MRI measures or cognitive decline.	Age, gender, presence of APOE ε4 allele, total energy intake, education, the time interval between completion of the FFQ and the measurement of the neuropsychological and MRI outcomes, BMI, physical activity, smoking status, cardiometabolic factors, and high level of depressive symptoms.	[117]
Cross-sectional and longitudinal	US	Adults from Boston Puerto Rican Health Study n= 1502 (378 M 1124 F) Age: 45–75 years at baseline	8 years	FFQ over 5-year follow-ups, neuropsychological tests, MMSE	Association between long-term adherence to MIND diet and cognitive function in Puerto Rican adults	In cross-sectional and longitudinal analyses, the highest compared to lowest MIND quintile associated with better cognition function, but not with cognitive trajectory over 8 years.	Income-to-poverty ratio, education level, and job complexity score.	[118]
Longitudinal study and meta-analysis	China	Participants from the China Health and Nutrition Survey (CHNS) n = 4066 participants Meta-analysis: results from the longitudinal study and 7 other MIND diet and cognitive effect studies n = 26,103	3-day 24 h dietary recall, battery of cognitive tests, immediate and delayed recall memory, attention, and calculation abilities	Median 3 years	Relationship between MIND diet and cognitive function and its decline among middle-aged and older adults	Higher MIND diet scores were significantly associated with better global cognitive function. Each increment of 3 points in MIND diet score has adjusted difference in global cognitive function z-score approximately equivalent to being one year younger in age. In the meta-analysis of 26,103 participants, 1 standardized deviation MIND score increment associated with 0.042-unit higher global cognitive function z-score and 0.014-unit slower annual cognitive decline.	Age, gender, education, annual household income per capita, residence, region, smoking, alcohol drinking, BMI, total energy intake, physical activity, history of chronic diseases included self-reported hypertension, diabetes, and myocardial infarction diagnosis, and use of antihypertensive and anti-diabetic medications.	[119]

Adapted from [20] and updated. AD: Alzheimer’s disease; APOE: apolipoprotein E; BMI: body mass index; CSIRO-FFQ: Commonwealth Scientific and Industrial Research Organization semi-quantitative food frequency questionnaire; EBMT: East Boston Memory Test; FFQ: Food Frequency Questionnaire; HDL: High-Density Lipoprotein; IADL: Instrumental Activities of Daily Living; MCI: mild cognitive impairment; MMSE: Mini-Mental State Examination; MRI: Magnetic Resonance Imaging; PA: high-intensity physical activity; PACC4: Preclinical Alzheimer’s Cognitive Composite 4; SBI: silent brain infarcts; SFFQ: semi-quantitative food frequency questionnaire; SMC: subjective memory complaints; TICS: Telephone Interview for Cognitive Status; ↘: decline; ↗: increase.

**Table 4:** A summary of clinical studies examining modified Mediterranean-ketogenic diet impact on ADRD.

Study Design	Country	Population	Follow-Up	Exposure	Outcome	Results	Covariates	Reference
Randomized, double-blind, crossover single-center pilot study	US	n = 17 Age: 64.6 ± 6.4 years Participants with MCI n = 11 Participants with normal cognitive function n = 6	6-week interventions: MMKD and AHA diet separated by 6-week washout	Gut microbiome, fecal short-chain fatty acids, markers of AD in cerebrospinal fluid	Change in gut microbial signature, fecal short-chain fatty acids, and AD biomarkers after each diet intervention.	MMKD associated with improved metabolic indices and AD biomarkers in CSF profile.	NS	[136]
Randomized crossover trial	US	Participants with MCI n= 9 (3 M 6 F) Age: 63.4 ± 4.0 years Participants with SMC n= 11 (2 M 9 F) Age: 64.9 ± 7.9 years	MMKD diet and AHA diet for 6 weeks followed by 6-week washout	Immediate and delayed memory, general cognition, plasma biomarkers, CSF biomarkers, and MRI	Compare effects of MMKD and AHA diet on AD biomarkers in CSF, neuroimaging measures, peripheral metabolism, and cognition.	MMKD associated with ↗ CSF Ab42 and ↘ t-tau.	Age, presence of APOE ε4 allele, and order of diet intervention.	[135]
Randomized controlled trial	New Zealand	Participants with diagnosed AD n= 26 (16 M 10 F) Age: 69.8 ± 6.0 years	12-week intervention and 10-week washout, low-fat diet and MMKD	FFQ, cognition by ACE-III, daily function by ADCS-ADL, quality of life by QOL-AD	Change in cognition, daily function, or quality of life in AD patients after 12 weeks of MMKD.	Improvement in cognitive function, everyday functioning, and quality of life for MMKD group participants compared with the low-fat diet ones.	NS	[137]
Crossover trial	US	Participants with MCI n = 9 (3 M 6 F) Age: 63.4 ± 4.0 Participants with SMC n = 11 (2 M 9 F) Age: 64.9 ± 7.89 years	MMKD and AHA diet for 6 weeks with a 6-week washout period	Dual-energy X-ray absorptiometry for adiposity and CSF biomarkers	Effects of MMKD compared to AHA diet on body weight, body composition, and body fat distribution and their association with AD biomarkers in CSF.	↘ in body fat on the MMKD related to changes in Aβ biomarkers.	Age and diet order	[134]

ACE-III: Addenbrookes Cognitive Examination-III; ADCS-ADL: Alzheimer’s Disease Cooperative Study—Activities of Daily Living; AD: Alzheimer’s disease; AHA: American Heart Association; APOE: apolipoprotein E; CSF: cerebrospinal fluid; MCI: mild cognitive impairment; MMKD: modified Mediterranean-ketogenic diet; MRI: Magnetic Resonance Imaging; PET: Positron Emission Tomography; SCFA: short-chain fatty acids; SMC: subjective memory complaints; QOL-AD: Quality of Life in Alzheimer’s Disease questionnaire; ↘: decline; ↗: increase.

**Table 5:** A summary of clinical studies examining vegetarian diet impact on ADRD.

Study Design	Country	Population	Follow-Up	Exposure	Outcome	Results	Covariates	Reference
NS	US and Canada	Vegetarian, n= 72 (32 M 40 F) (62 white, 10 non-white) Age: 76.6 ± 8.3 years Non-vegetarian, n = 60 (24 M 36 F) (44 white, 16 non-white) Age: 73.5 ± 7.7 years	10 years	FFQ, historic dietary habits survey instrument	Verbal learning, memory, attention, processing speed, executive function, visuospatial abilities, language, global cognitive functioning, MMSE, AMNART, and GDS	Vegetarian diet not associated with lower mild memory impairment likelihood. More stable diet associated with better memory/language.	Age, education	[143]
Prospective cohort study	Taiwan	Buddhist Tzu Chi Foundation volunteers Non-vegetarian, n = 3154 (1345 M 1809 F) Age: 57.8 ± 6.3 years Vegetarian, n = 1737 (461 M 1276 F) Age: 58.1 ± 6.5 years	Average 9.2 years	Baseline questionnaires to assess dietary pattern, overall plant-based diet index	MCI and dementia diagnosis	Vegetarians associated with less risk of dementia compared with non-vegetarians after adjusting for covariates.	Gender, age, smoking, drinking, education level, marital status, regular exercise, comorbidities	[144]
Longitudinal	US	n = 3337 (1200 M 2137 F) Age: 73.7 ± 5.72 years African American (AA), n = 2012; white, n = 1325	NS	FFQ	Episodic memory, perceptual speed, and MMSE	↗ plant-based diet associated with slower ↘ in GC, PS, and EM in AA but not in white participants. AA in the highest quintile of high plant-based diet had significantly slower rates of GC, PS, and EM than individuals in the lowest quintile.	Age, gender, presence of apolipoprotein E4 (APOE ε4) allele, lifestyle factors (education, cognitive activities, smoking, calorie intake, risk factors for cardiovascular disease, time and interaction time × each covariate)	[141]

AMNART: American National Adult Reading Test; APOE: apolipoprotein E; EM: episodic memory; FFQ: Food Frequency Questionnaire; GC: global cognition; GDS: Geriatric Depression Scale; MCI: mild cognitive impairment; MMSE: Mini-Mental State Examination; NS: not specified; PS: perceptual speed; VIQ: estimated verbal intelligence; ↘: decline; ↗: increase.

## Data Availability

Not applicable.
